# New Drugs, Appliances, and Things Medical

**Published:** 1893-01-28

**Authors:** 


### NEW DRUGS, APPLIANCES, AND THINGS MEDICAL.

[All preparations, appliances, novelties, etc., of which a notice is desired, should be sent for the Editor, to care of The Manager, 140, Strand, London, W.C.]

### AN ADMIRABLE HOT-WATER BOTTLE.

Few are insensible to the comfort a hot-water bottle confers, and none are wise who venture between the sheets, when travelling in winter, without the safeguard of such a companion. Since the happy introduction of that admirable commodity, the indiarubber bottle, all the objections to the use of hot-water bottles has been removed. There is one point in respect to the rubber goods which should be especially attended to, and that is, that the quality should be good. No economy is attained by using aught but the best. Those in search of a really first-rate article, we would recommend to send for one of Messrs. Anderson, Anderson, and Anderson's bottles, feeling sure, from the specimens we have seen of their goods, that they cannot possibly do better. The rubber is of first-rate quality, soft and pliable, but sufficiently thick also for durability. They are supplied with funnel tops, which are excellently finished. The prices are most moderate, the first size costing 3s. 10d., and the largest 8s. 9d. Five per cent. discount for cash brings the purchase still lower. Covers are provided from 1s. Messrs. Anderson's address is 37, Queen Victoria Street, E.C.

